# Lectin-based carbohydrate profile of megakaryocytes in murine fetal liver during development

**DOI:** 10.1038/s41598-023-32863-3

**Published:** 2023-04-25

**Authors:** Barbara Cristina Marcollino Bomfim, Jessyca Azevedo-Silva, Giulia Caminha, João Paulo Rodrigues Santos, Marcelo Pelajo-Machado, Jackline de Paula Ayres-Silva

**Affiliations:** 1 Laboratory of Pathology, Oswaldo Cruz Institute - Oswaldo Cruz Foundation (Fiocruz), Rio de Janeiro, Brazil; 2 National Institute of Science and Technology on Neuroimmunomodulation (INCT-NIM), Oswaldo Cruz Institute, Oswaldo Cruz Foundation (Fiocruz), Rio de Janeiro, Brazil

**Keywords:** Cell biology, Histology, Immunohistochemistry, Confocal microscopy, Developmental biology, Haematopoiesis, Thrombopoiesis, Carbohydrates, Glycobiology, Histocytochemistry

## Abstract

Hematopoiesis is the process by which blood cells are generated. During embryonic development, these cells migrate through different organs until they reach the bone marrow, their definitive place in adulthood. Around E10.5, the fetal liver starts budding from the gut, where first hematopoietic cells arrive and expand. Hematopoietic cell migration occurs through cytokine stimulation, receptor expression, and glycosylation patterns on the cell surface. In addition, carbohydrates can modulate different cell activation states. For this reason, we aimed to characterize and quantify fetal megakaryocytic cells in mouse fetal liver according to their glycan residues at different gestational ages through lectins. Mouse fetuses between E11.5 and E18.5 were formalin-fixed and, paraffin-embedded, for immunofluorescence analysis using confocal microscopy. The results showed that the following sugar residues were expressed in proliferating and differentiating megakaryocytes in the fetal liver at different gestational ages: α-mannose, α-glucose, galactose, GlcNAc, and two types of complex oligosaccharides. Megakaryocytes also showed three proliferation waves during liver development at E12.5, E14.5, and E18.5. Additionally, the lectins that exhibited high and specific pattern intensities at liver capsules and vessels were shown to be a less time-consuming and robust alternative alternative to conventional antibodies for displaying liver structures such as capsules and vessels, as well as for megakaryocyte differentiation in the fetal liver.

## Introduction

The ontogeny of hematopoiesis in mammals is a long process coordinated by several transcription factors and migratory molecules that promote differentiation of hematopoietic stem cells into various lineages at each embryonic hematopoietic site^1–3^. In mice, these locations are divided into extraembryonic, between gestational ages E7.25 to E10.5, such as the yolk sac and placenta^4^, and intraembryonic, starting at E10.5, such as the aorta-gonads-mesonephros (AGM) region, liver, spleen, and bone marrow, starting at E16.5^3,5^.

One of the most important temporary sites of definitive hematopoiesis is the fetal liver, which is a complex and favorable organ for the expansion, differentiation, and maturation of myeloid and erythroid lineages originating from the yolk sac and AGM region^6–8^. Four stages of fetal liver hematopoiesis starting from E10.5 were described when the fetal liver is colonized by immature hematopoietic cells for the first time, showing nucleated red blood cells coming through the blood circulation that allocate the hepatic sinusoids^9,10^. Stage II describes the expansion of the hematopoietic compartment from E11.5 − 12.5. With liver development, around E13.5, hepatocytes support the differentiation of myeloid lineage cells, like granulocytes, monocytes, and megakaryocytes, providing a unique environment which supports temporary hematopoiesis, before the bone marrow is formed and able to sustain it^2,11,12^. Hematopoietic expansion starts declining in the liver from E15.5, due to the initiation of hematopoietic cell migration to the bone marrow^9,10^. However, the liver still presents an extensive number of hematopoietic cells by E16.5^11^, coinciding with the passage of erythropoiesis in the fetal spleen, which is also a transitional organ acting as a bridge between the fetal liver and the bone marrow during development^2^. In adult life of mammals, bone marrow is the main hematopoietic organ, however the spleen and liver can also partake of hematopoiesis in humans^13,14^ and adult mice^15,16^.

The mechanisms responsible for hematopoietic cells homing during ontogeny and their interaction with endothelial cells are still not known in depth. Thus, some tools have been used to identify the molecules involved during homing of hematopoietic cells, such as integrins, selectins, and cytokines^1^. Among them, the detection of relevant sugars to the interaction between hematopoietic and endothelial cells have been adopted, such as lectin labellings and the removal of sugar residues from hematopoietic cells to identify those molecules involved in the cell migration. For example, galactosyl and mannosyl residues have been described in the migrant cell-endothelium interaction process^17,18^ and sialic acid residues during cell migration to the bone marrow^19,20^.

Lectins are proteins, glycoproteins and/or glycoconjugates of non-immune origin, with the ability to specifically recognize and/or precipitate substances or cells containing carbohydrates without changing the covalent structure of glycosidic ligands^21^. Lectins enable detailed visualization of tissue morphology, depending on the type of sugar expressed there, serving as markers of cell development stages consisting of a cheaper and faster strategy than antibodies^22^. Lectin-based carbohydrate profiling of cells, tissues and organs may reveal varied biomarkers according to different developmental stages from embryonic to adult life in both physiological and pathological, conditions, identifying even disease-specific stages^23^. The glycosylation pattern of tumor cells is also altered, and lectins have been widely used for studying and marking such alterations^24–27^.

Megakaryocytes are cells described as present since the yolk sac, responsible for homeostasis control. Despite robust knowledge available about platelets and coagulation, especially concerning their central role in homeostasis and diseases, gaps are still found in megakaryocyte development^28^. Knowing how those cells develop, the genes involved in the differentiation process, and how the expressed molecules modulate hematopoietic niches is crucial for understanding physiopathological process involving such cells^29,30^. Some studies have pointed out differences between adult and fetal megakaryocytes^31,32^, which demonstrates the importance of a better characterizing and understanding of the differentiation and homing processes during megakaryocyte development, especially during fetal stages^20,21^. This knowledge gap in which sugars residues are involved in the megakaryocytes differentiation and homing process during fetal development^29–32^, prompted us to characterize the lectin-based carbohydrate residues from megakaryocytes in the fetal livers of mice at different gestational ages, as well as the glycan residues expressed on the surface of other liver structures such as capsules and vessels. This study shows for the first time spatio-temporal changes on carbohydrates present in mouse fetal liver and megakaryocytes, enhancing fundamental knowledge on this field of research.

## Results

### *Lens culinaris* agglutinin (LCA), *Pisum sativum* agglutinin (PSA) and Concanavalin A (CON A) (α-mannose, α-glucose)

Concanavalin A (CON A) is a lectin extracted from the jack-bean (*Canavalia ensiformis*) legume, classified as a L-lectin found in seeds, which recognizes α-mannose, α-glucose sugar residues. It has several applications in the laboratory such as a mitogen, especially for T cells. *Lens culinaris* lectin/agglutinin (LCA) is extracted from lentil seeds, is widely used in the feeding of many species, especially humans and recognizes mostly α-mannose. *Pisum sativum* (PSA) is extracted from peas and recognizes α-mannose.

**Figure 1:**
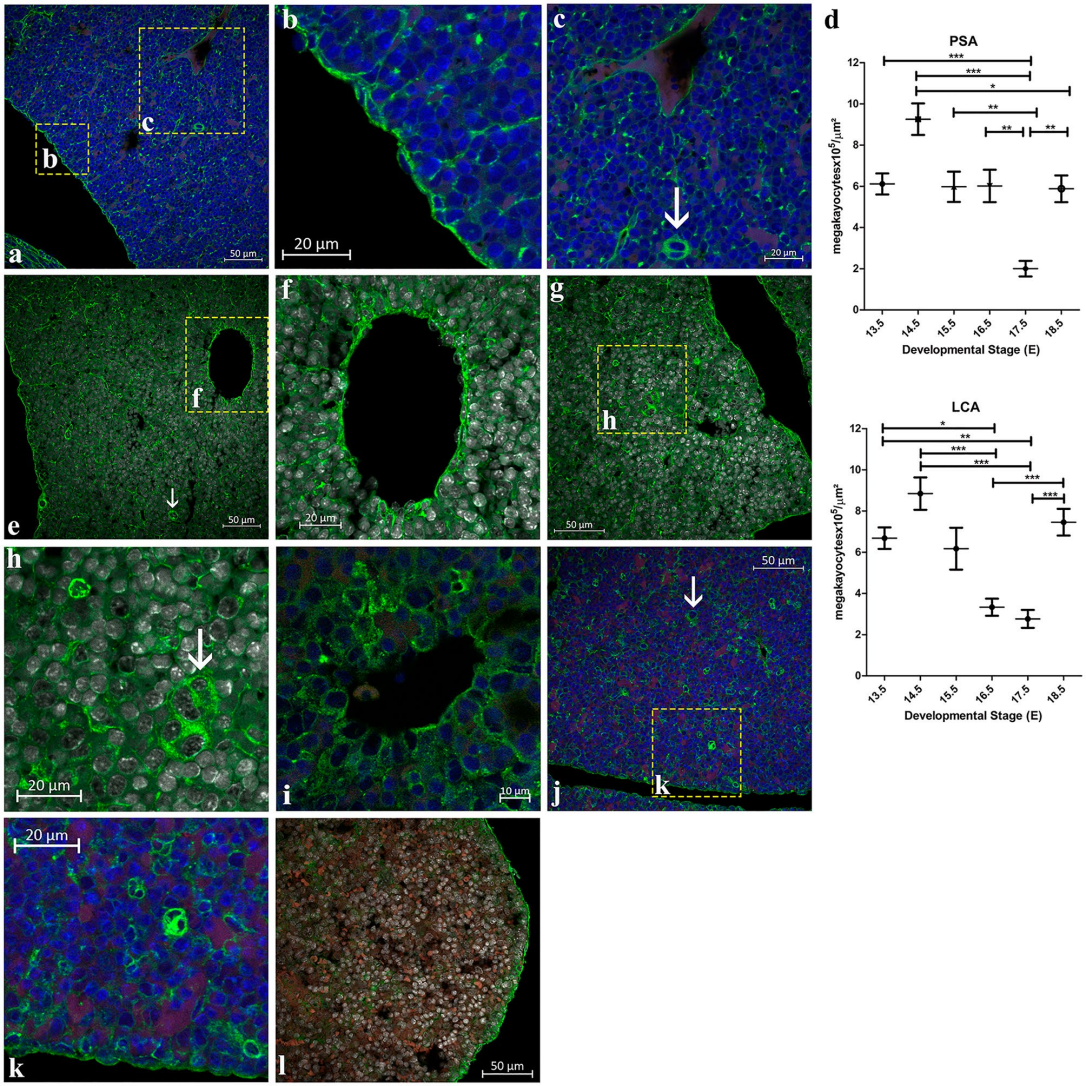
Mannose and glucose expression revealed by LCA (**a–c**), PSA (**e–h**) and CON A (**i–l**) lectins. An average of five fetal livers per slide from E13.5 (**i**), E15.5 (**a–c,j,k**), E16.5 (**e–h**) and E18.5 (**l**) were analyzed in two independent experiments and representative images are shown. Capsule and vessels intensely labeled, as well as megakaryocytes (arrow). D – graph with LCA and PSA *in situ* quantification of megakaryocytes. Results are presented as means and error bars denote ±SEM. At LCA experiment, an average of 21(16-30) fields were counted (mean-min-max) from E13.5 to 18.5. At PSA experiment, an average of 26 (16-46) fields were counted (mean-min-max) from E13.5 to 18.5. Green – AlexaFluor 488; white or blue (pseudocolors) – DAPI; red – autofluorescence.

*Lens culinaris* agglutinin (LCA) (Fig. 1a–c) and *Pisium sativum* (PSA) (Fig. 1e–h) showed intense labeling of the capsule in the fetal liver starting at the gestational age of E11.5 (Tables 1, 2), in addition to other small cells in the hepatic parenchyma, probably from the hematopoietic lineage, (Fig. 1g,h). Central portal veins were uniformly and intensely labeled throughout all gestational ages analyzed, probably restricted to endothelial cells, in contrast to a discrete expression in small vessels in the hepatic sinusoids from E13.5-E18.5. Megakaryocytes showed different intensities of staining for mannose and glucose according to the gestational ages investigated, but in general, they revealed high intensity of these residues; therefore, we used these lectins for *in situ* quantification of megakaryocytes during fetal liver development (Fig. 1d). In early fetal liver, hepatocytes remain in the differentiation process and are not yet suitable for the expansion and differentiation of hematopoietic cells. Therefore, the fetal liver from E11.5 to E13.5 was small and lodges fewer hematopoietic cells, although a first wave of megakaryocyte migration to the liver at E14.5 were noticed. At E15.5 − 16.5 was observed a decline in the number of megakaryocytes per field until the end of the *in-uterus* development with the two lectins used, LCA and PSA, and a third small peak at E18.5 (Fig. 1d).

**Table 1:** Comparative table of lectins utilized: Evidenced structures and fetal liver cells.

	Lectins	Megakaryocyte	Capsule	Vessels
Mannose and glucose	CON A	+	+	+
	LCA	++	++	++
	PSA	++	++	++
Galactose	PNA	−	−	−
	APL	+	+	+
	JACALIN (AIL)	+	+	+
	ECL	+	++	+
	RCA	+	+	−
	GSL I	−	−	+/−
Oligosaccharide	PHA-E	++	++	+
	PHA-L	+	−	+
N-Acetylglucosamine	DSL	−	+/−	+/−
	sWGA	+	+	+
	WGA	+	++	++
	STL	+	+	+/−
	GSL II	−	−	+
	LEL	+	+	−
N-Acetylgalactosamine	SBA	−	−	−
	DBA	−	−	−
	VVA	−	−	−
Fucose	UEA	−	−	−

+ Expressed, ++ intensely expressed, +/− Expressed at some developmental time points, − not expressed.

**Table 2:** Semi quantitative estimation of glycan expression through lectin labeling in megakaryocytes: Legend: G.A. gestational age.

G.A.	Mannose and Glucose			Galactose						Oligosaccharide	
	CON A	PSA	LCA	PNA	APL	JACALIN (AIL)	ECL	RCA	GSL I	PHA-E	PHA-L
11. 5	−	++	++	nt	nt	+	nt	−	nt	+	−
12. 5	−	++	++	nt	nt	+	nt	−	nt	−	+
13. 5	−	++	++	−	++	−	+	−	−	++	+
14. 5	+	++	++	−	+	+	+	++	−	++	++
15. 5	++	++	++	−	+	++	+	−	−	++	++
16. 5	−	++	++	−	+	++	+	++	−	++	++
17. 5	−	++	++	nt	nt	+	nt	−	nt	+	+
18. 5	−	++	++	nt	nt	+	nt	−	nt	+	+

G.A.	N-Acetylglucosamine										
	DSL	sWGA	WGA	STL	GSL II	LEL					
11. 5	nt	nt	−	nt	nt	nt					
12. 5	nt	nt	−	nt	nt	nt					
13. 5	−	−	++	+	−	+					
14. 5	−	++	++	+	−	+					
15. 5	−	−	−	+	−	+					
16. 5	−	−	+	+	−	+					
17. 5	nt	nt	−	nt	nt	nt					
18. 5	nt	nt	−	nt	nt	nt					

(+) expressed, (++) intensely expressed, (−) not expressed, *nt* not detected.

Likewise, the lectin Concanavalin A (ConA) (Fig. 1i–l) revealed an intensely labeled capsule from all gestational ages analyzed (Fig. 1i–k), in addition to a sharper fluorescence compared to LCA and PSA. In contrast, megakaryocytes showed scarce amounts of mannose and glucose from E11.5 − E13.5, followed by a short temporal expression observed from to E14.5 − 15.5, of high intensity (Fig. 1j), and a quick decrease to E18.5 (Fig. 1l). Other cells in the liver parenchyma were also labeled by ConA in their cytoplasm, and some showed high-intensity labeling presenting morphological characteristics resembling those of granulocytes (Fig. 1k). Central vessels started expressing mannose and glucose at E13.5 to E15.5, presenting a dotted pattern (Fig 1i), with a polarization to endothelial cell surfaces in contact with the lumen (Fig. 1e), but not restricted to them when Con A was applied.

### Peanut agglutinin (PNA) *Arachis hypogaea *Agglutinin,* Abrus precatorius *lectin (APL), *Artocarpus integrifolia *lectin (Jackfruit) Jacalin, *Erythrina cristagalli* lectin (ECL), *Ricinus communis* Agglutinin (RCA 120), *Griffonia simplicifolia* lectin I (GSI) (galactose)

*Arachis hypogea* lectin (PNA) is extracted from peanuts, and binds preferentially to T-antigen, a galactosyl (β-1,3) N-acetylgalactosamine structure present in many glycoconjugates such as M and N blood groups, gangliosides, and many other soluble and membrane-associated glycoproteins and glycolipids, and also recognizes galactose. *Abrus precatorius* lectin (ABL) is extracted from a flowering plant commonly known as jequirity bean or rosary pea, which recognizes galactose residues. *Artocarpus integrifolia* lectin, known as jacalin, is extracted from the jackfruit, mostly found in tropical forests and very popular in India, Asia and Brazil. This lectin acts as a T cell mitogen and is used to purify human IgA. Jacalin is a lectin composed of four subunits of approximately 16 kDa each, which appears to bind only O-glycosidically linked oligosaccharides, preferring the structure galactosyl (β-1,3) N-acetylgalactosamine. Erythrina cristagalli (ECL) is a flowering tree known as cockspur coral, native from South America, also very popular in California. ECL lectin recognizes Galactosyl β4 N-acetylgalactosamine, galactose and lactose residues and is used to negatively select NK cells. Ricinus communis (RCA 120) is a perennial flowering plant known as castor bean or castor oil plant that binds to galactose, N-acetylgalactosamine and lactose residues. This lectin consists of two subunits of 60 kDa which can be dissociated by 27 and 33 kDa. *Griffonia simplicifolia*, (GSI/BSI) previously named as *Bandeiraea simplicifolia*, is a woody climbing shrub native of West Africa and Central Africa. The lectin extracted from this plant binds to galactose and N-Acetylgalactosamine residues. GSI is a lectin of 114 kDa composed by two types of subunits (A and B) that combines forming tetrameric structures, thus resulting in five isolectins.

We tested six lectins described to recognize galactose residues in fetal livers from to E13.5 − 16.5, and five of them bound in the vessel walls of different diameters, liver capsules, and megakaryocytes of different sizes (Table 2). We extended the study from to E11.5 − 18.5 applying jacalin, the lectin that most effectively marked megakaryocytes in the limited temporary screening corresponding to the highest hematopoietic activity in the fetal liver.

**Figure 2:**
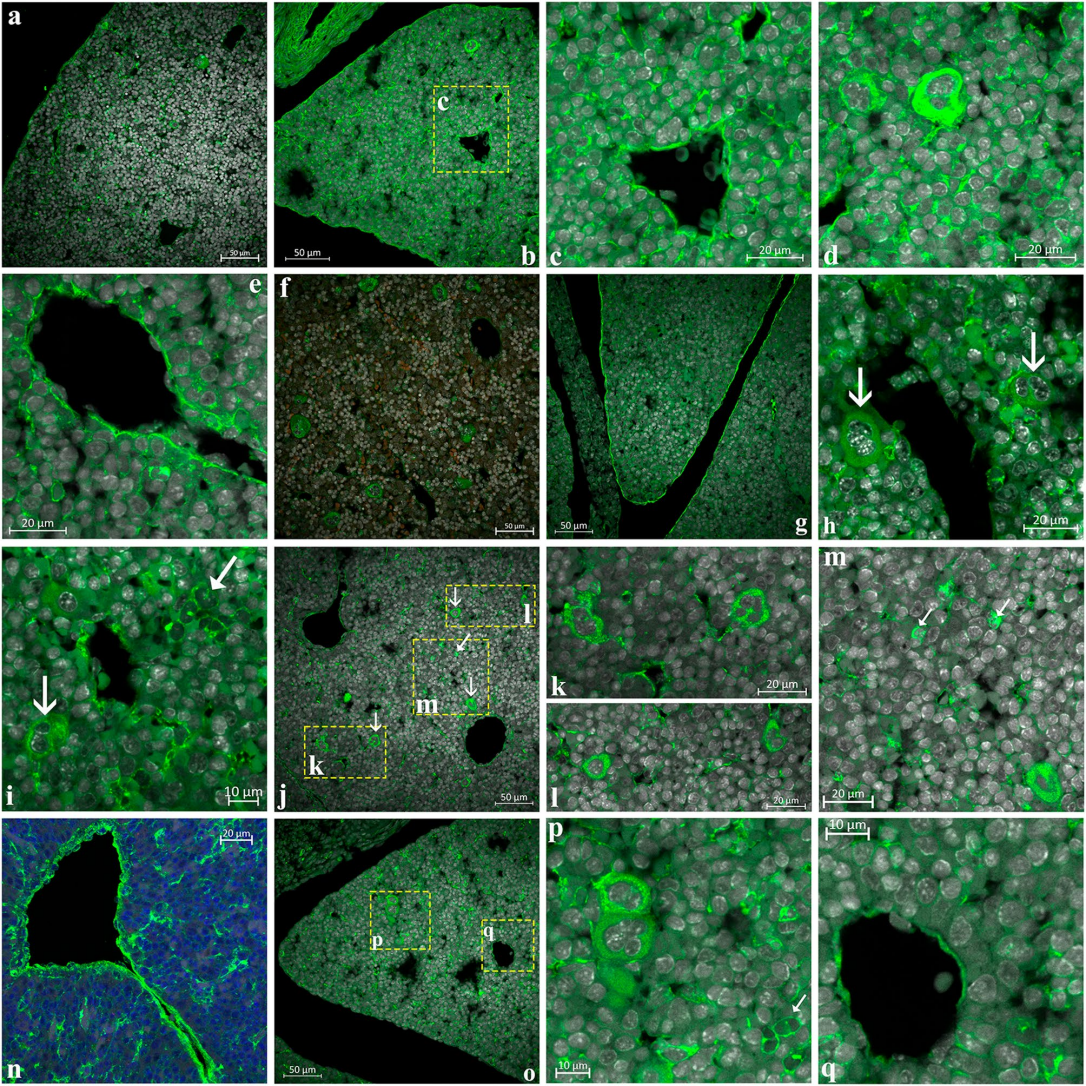
Galactose expression revealed by APL (**a**), Jacalin (AIL) (**b–e**), ECL (**f**), RCA (**g–i**), and complex galactose residues by PHA-E (**j–n**) and PHA-L lectins (**o–q**). An average of five fetal livers per slide from E15.5 (**f,n**) and E16.5 (**a–e,g–m,o–q**) were analyzed in two independent experiments and representative images are shown. Vessels and megakaryocytes (arrow) strongly marked by different galactose residues. Smaller cells of horseshoe shaped nucleus that resembles neutrophils, band stage (triangle point arrow) at (**i,j,m,p**). Green—AlexaFluor 488; white or blue (pseudocolors)—DAPI.

APL showed intense galactose residues in megakaryocytes and endothelial cells from sinusoids at E13.5-E16.5 (Fig. 2a). Jacalin marked megakaryocytes from E12.5, presenting higher intensity at E16.5 (Fig. 2b–e), and a decreasing to E18.5. From E13.5, the central veins and sinusoids were weakly labeled, showing an increase in the intensity proportional to the advancement of the gestational age up to E16.5 (Fig. 2b,c,e), when decrease started. Capsules were labeled at all ages, showing higher expression at E16.5. A dotted pattern of galactose was observed from E13.5 − 16.5, probably from the hematopoietic lineage in cells in the liver parenchyma. ECL marked intensly the capsule at E14.5, whilst megakaryocytes were less bright showing a punctuated pattern. The lectin-marked vessels were faintly noticeable (Fig. 2f). RCA weakly exhibited galactose residues in the capsule from E11.5, higher than E14.5, until near birth at E18.5. The megakaryocytes were evident from E14.5 onwards (Fig. 2g–i), and other hematopoietic cells, putatively neutrophils, were also highly labeled at E16.5 (Fig. 2i). The sinusoids were negative for RCA binding (Fig. 2h,i). GSI lectin labeled hematopoietic cells, morphologically resembling neutrophils, from E13.5 − 15.5. Vessel walls were labeled weakly with GSI and a decrease in intensity was observed after E16.5 when it turned negative. PNA did not show any labeling in the liver, and other fetal organs, such as the gut and pancreas.

### *Phaseolus vulgaris* eritroagglutinin (PHA-E) (Oligosaccharide: (Gal4GlcNAc2Man6 (GlcNAc4) (GlcNAc4Man3) Man4)) and* Phaseolus vulgaris* leucoagglutinin (PHA-L) (Oligosaccharide: Gal4GlcNAc6 (GlcNAc2Man3) Man3))

*Phaseolus vulgaris* is an herbaceous plant commonly known as beans. From this plant a family of lectins which consists of four subunits is extracted, one of them is named E for erythroagglutinin and L for leucoagglutinin, involved in the red and white blood cell agglutination. The combination of these subunits produces five isolectins, and bounds to galactose and complex sugar residues structures (Supplementary Table S1).

It was noted that PHA-E recognized several structures, such as the capsule, central veins, sinusoids, and megakaryocytes, with higher intensity in the last two, in all ages analyzed, due to its broad glycan spectrum recognition (Fig. 2i–n). In contrast, PHA-L, which also reveal complex glycans, weakly marked the central veins until E12.5, gaining greater expression of oligosaccharides from this age onwards, presenting a higher intensity at E15.5 in the sinusoids (Fig. 2o–q), when started to decrease to E18.5. Megakaryocytes showed less intense labeling at E13.5, with an increase from E15.5 − E16.5 (Fig. 2p). Other small cells were observed in the hepatic parenchyma, showing small clusters starting at E13.5, which became higher in size and intensity (Fig. 2p). Capsules were negative at all ages analyzed (Fig. 2o).

### *Datura stramonium* lectin (DSL), (succinylated) Wheat germ agglutinin (sWGA/WGA): *Triticum vulgaris *(N-acetylglucosamine/sialic acid), *Solanum tuberosum* lectin (STL) (N-acetylglucosamine), *Griffonia simplicifolia* lectin II (GSLII), *Lycopersicon esculentum* lectin (LEL)

*Datura stramonium* (DSL) is a poisonous flowering plant knowing as thorn apple, devil’s snare or devil’s trumpet originally from Central America. This plant recognizes (β-1,4) linked N-acetylglucosamine oligomers, preferring chitobiose or chitotriose over a single N-acetylglucosamine residue. Wheat germ agglutinin (WGA) is a lectin extracted from *Triticum vulgaris* that binds N-acetylglucosamine, mostly found in insects’ chitin, cell membrane of yeast and bacteria, cartilage, inner nose, and cornea of mammals. WGA preferentially bounds to dimers and trimers of N-acetylglucosamine, also binding to sialic acid residues while succinylated WGA does not bind to sialic acid residues. *Griffonia simplicifolia* lectin II (GSII) previously named as *Bandeiraea simplicifolia*, recognizes exclusively α- or β-linked N-acetylglucosamine residues at the nonreducing terminal of oligosaccharides. *Lycopersicon esculentum* (LEL), also known as *Solanum lycopersicum*, is the tomato plant and their lectin recognizes [GlcNAc]1-3, N-Acetylglucosamine, mostly used to show blood vessels and microglial cells in rodents.

**Figure 3:**
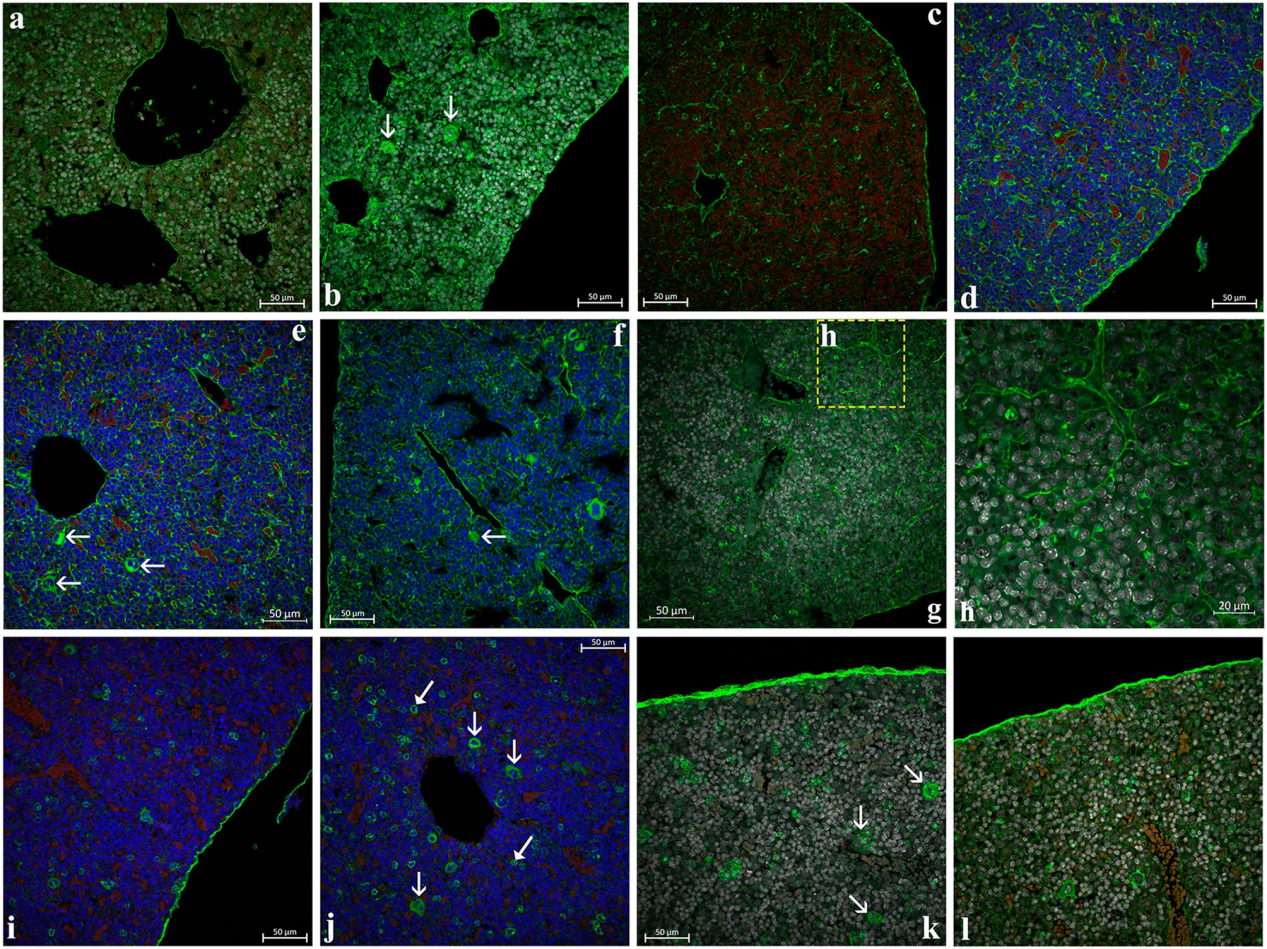
N-acetylglucosamine expression revealed by DSL (**a**), sWGA (**b,c**), WGA (**d–h**), STL (**i–k**), LEL (**l**). An average of five fetal livers per slidefrom E13.5 (**a,d-e,i–j**), E14.5 (**b,f,k,l**), and E16.5 (**c,g–h**) were analyzed in two independent experiments and representative images are shown. Megakaryocytes (arrow) intensely visualized by sWGA, WGA and STL. Capsule strongly express NAcGlu marked through STL. Some smaller cells of horseshoe shaped nucleus, probably neutrophils at band stage (triangle point arrow, fig (**j**)). Green—AlexaFluor 488; white, blue or red (**c**) (pseudocolors)—DAPI; red (**d,e,i,j**)—autofluorescence.

On E13.5, DSL marked vessels and capsules with a delineated and of a delicate pattern (Fig. 3a). Between E13.5 and E14.5, vessels were observed with a greater affinity than for the capsule. At E15.5, a slight stamp on the vessels and capsule with a delineated pattern was observed. After E16.5, all structures revealed by DSL at the previous gestational ages were negative.

The sWGA lectin showed expression of N-acetylglucosamine in endothelial cells and capsule from E13.5-16.5 (Fig. 3b,c). Megakaryocytes were in evidence only at E14.5 (Fig. 3b). The WGA lectin demonstrated glycan residues in the capsule in all ages analyzed (Fig. 3d,f,g). Central veins and intrahepatic sinusoids were strongly evidenced by WGA at E14.5, which proved to be an excellent marker (Fig. 3f). In contrast, the recognition of GlcNAc and Sialic Acid in megakaryocytes was faint, with a delicate layer on the membrane surface at all ages analyzed. At E13.5-14.5, these glycans were intensely observed inside the cytoplasm (Fig. 3e,f). Other cells in the hepatic parenchyma, likely from the hematopoietic lineage, showed higher expression of WGA lectin from to E13.5-E16.5 (Fig. 3d,e,h), with a decrease in the later gestational ages. The STL lectin showed a capsule and megakaryocytes from E13.5-16.5 (Fig. 3i–k). At E14.5, an increase of N-acetylglucosamine expression in the capsule’s layer was observed (Fig. 3k), as well as an expansion in the number of hematopoietic cells positively bind to STL (Fig. 3j–k), probably from a neutrophilic lineage. Vessel walls were slightly positive from E13.5 − 15.5. After 15.5 the reactivity to STL disappeared. GSL II labeled only portal vessels of a discontinuous pattern, along with some hematopoietic cells that require further characterization at E 13.5. We applied LEL in fetal livers from E13.5 − 15.5, showing capsules, megakaryocytes, and other hematopoietic cells that require deeper characterization (Fig. 3l).

### Soybean agglutinin (SBA), *Dolichos biflorus* agglutinin (DBA), *Vicia villosa* Agglutinin (VVA), Glycine max (GM) (N-acetylgalactosamine)

Soybean agglutinin or lectins (SBA/SBL) are extracted from soybeans (*Glycine max*) with a 120kDA which preferentially binds to oligosaccharide structures presenting terminal α- or β-linked N-acetylgalactosamine, and to a lesser extent, galactose residues. *Vicia vilosa* agglutinin (VVA) is extracted from seeds of hairy, fodder or winter vetch, a plant native from Europe and Western Asia. This lectin preferentially binds to α- or β-linked terminal N-acetylgalactosamine, especially a single α-N-acetylgalactosamine residue linked to serine or threonine in a polypeptide.

Lectins that recognize N-acetyl galactosamine were negative to the vessels and capsule from the fetal liver and to proliferating, and differentiating hematopoietic cells inside this organ from E13.5 − 16.5 (Table 1). Other fetal structures were positive and served as internal controls such as the pancreas and intestine.

### Ulex europaeus agglutinin (UEA-I) (Fucose)

*Ulex europaeus* is a flowering plant known as gorse, furze or whin, originally from the British Isles and Western Europe that binds to many glycoproteins and glycolipids containing α-linked fucose residues, such as ABO blood group glycoconjugates, with especial attention to the O group of blood cells. It is also an excellent marker for human endothelial cells.

This lectin was negative in the fetal liver, as observed with lectins that recognize N-acetylgalactosamine, in contrast to some intestinal cells that served as positive internal controls (Table 1).

### Von Willebrand factor (vWF)

**Figure 4:**
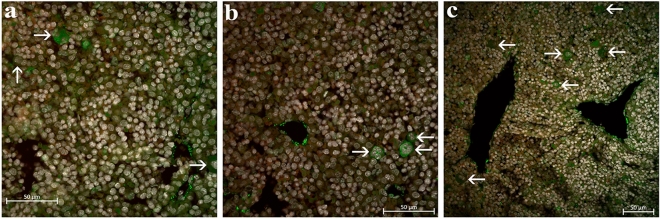
vWF expression at five fetal livers from E14.5.Two independent experiments were carried out and representative images are shown. Dotted expression in endothelial cells from portal vessels and different intensities inside from smaller to large size megakaryocytes (arrow). Green – AlexaFluor 488; white – DAPI.

We applied the vWF antibody to show fetal liver vessels and megakaryocytes, and the results were less intense than those obtained by lectins, which labeled those two structures more effectively, especially mannose and glucose residues (Fig. 4a–c). The measurement of megakaryocytes diameters in 16 cells from 3 different images was 15,015 ± 2690 μm (mean ± standard deviation) what demonstrated that those cells are smaller than those observed in the bone marrow.

To summarize, the labeling of megakaryocytes was observed for 13 lectins, with mannose, glucose, galactose, and oligosaccharides being the most abundant glycan residues and a small contribution of N-acetylglucosamines. LCA and PSA revealed this lineage in all ages analyzed. The liver capsule was also observed using LCA, PSA, ECL, PHA-E, and WGA. In contrast to DSL, which revealed the capsule until E16.5, PNA and GSL II could not unveil the capsule. The central portal vessels and sinusoids were labeled by 14 lectins, except for PNA, RCA, and LEL. DSL and STL did not detect endothelial cells after E16.5. N-acetylgalactosamine is not expressed in the capsule and vessels during fetal liver development, as we tested three lectins that recognize this sugar, and none of them were positive from E13.5 − 16.5. Tables 1 and 2 show the lectin labeling patterns observed in fetal liver structures and megakaryocytes at different gestational ages analyzed. Supplementary Fig. S4 shows morphological description of megakaryocytes at different stages of fetal liver development.

## Discussion

Our results showed the staining of at least five different glycan residues in the developing structures of the fetal livers, such as the capsule and vessels, as well as in hematopoietic cells through mouse development. At least 13 lectins were differentially bound in fetal liver megakaryocytes at several intensities and at different developmental ages. Such differences may be related to the glycan recognition structure of the lectin as well as the cell differentiation process when some lectins present more affinity at determined gestational ages and cell populations.

Previous studies have reported the labeling of LCA and PSA in megakaryocytes, granulocytes, and other hematopoietic cells as well as the negative pattern of Con A for most cell types, except for the granulocytic lineage^33,34^. However, there are few reports of Con A labeling in human platelet membranes and/or megakaryocytes^35,36^. Membrane platelets seem to have a broad sugar expression spectrum correlated with their metabolic activation. At megakaryocytes, lectin binding could differ depending on the morphology and differentiation stage^22,33,34^.

**Figure 5:**
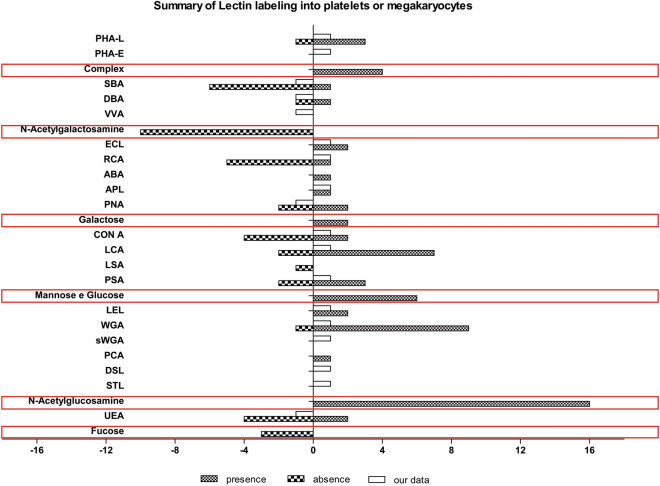
Summary of literature review from Suppl. Table S2 and results from this study, assembling data from humans, mouse, cats, dogs, guinea pigs from 21 lectins, also clustered by sugar recognition type as complex (PHA-E and PHA-L), N-acetylgalactosamine, galactose, mannose and glucose, N-acetylglucosamine and fucose. These clustering bars represent the final score from total absence (negative score) and presence (positive score) related.

We summarized the literature results using lectins to recognize platelets or megakaryocyte molecules in many models (humans, guinea pigs, cats, and dogs), holding a total of 16 lectins, two of them showing negative labeling for megakaryocytes: CON A and RCA (Suppl. Table S2). Nevertheless, SBA and UEA were negative in cats and dogs and positive in humans, indicating differences among species. Compared to platelets, CON A, RCA, and UEA were positive in humans and negative in cats and dogs, whereas SBA, DBA, PNA, and LSA were negative in the species tested. Interestingly, when analyzing all lectin data together, including platelets, megakaryocytes, and all species, most lectins capable of recognizing N-acetylgalactosamine residues alone or together were negative, while mannose, glucose, and N-acetylglucosamine residues were more likely to be present, with the most employed and more robust pattern addressed to LCA and WGA. N-acetylglucosamine is reported as a lectin-based carbohydrate residue always present, with two other previous lectins applied in different studies being positive in platelets or megakaryocytes. In our study, we also observed three more lectins that recognize the same sugar with similar patterns (Fig. 5).

WGA lectin also recognizes sialic acid of less intensity and N-acetylglucosamine residues, and previously reported to recognize megakaryocytes from different species, such as cats, dogs, guinea pigs, and humans^22,33,34^. Schick and Filmyer^22^ demonstrated that approximately half of adult bone marrow megakaryocytes from guinea pigs are positive for WGA, in which the morphological stage is more determinant for high positivity than ploidy and size^22^. They observed that megakaryocytes at morphological differentiation stages III and IV were more likely to express sialic acid residues with almost 70%, in contrast to approximately 30% of those at stages I and II. However, Darbès et al. observed no clear correlation between the maturational stage and staining of megakaryocytic cells from cats and dogs with WGA^34^. In our study, we detected WGA-binding residues in most fetal megakaryocytes from E14.5 − 16.5. At this stage the fetal liver is large and harbor many hematopoietic cells at several differentiation stages. Before this gestational age, megakaryocytes were possibly not completely differentiated, and after E16.5, those cells migrated out of the fetal liver. This lectin was not able to efficiently label immature megakaryocytes in the fetal liver, such as PSA and PNA.

Nachman et al. isolated human platelet membranes and examined them after elution through lectin affinity chromatography using Con A and detected a glycoprotein of ~100kDa (named gpI) of the same molecular weight as the major platelet surface glycoprotein^37^. Greenberg and Jamieson assayed platelets and membrane platelets against nine lectins (WGA, RCA, LCA, PCA, ABA, Con A, LSA, PSA, and SBA), and observed aggregation and/or agglutination effects when WGA, RCA, PCA, ABA, and Con A were applied^35^. They also characterized agglutination inhibition when gpI was employed in the presence of WGA, GpII (22.5 kDa) agglutination inhibition against PCA, and gpIII (5kDa) did not inhibit agglutination in any of the nine lectins tested^35^. Clemetson et al. used LCA, WGA, and APL lectins to purify membrane glycoproteins (gp) from human blood platelets, and obtained LCA binding to gp Ia, IIb, and part of IIIa. WGA binds to Ia and Ib, and APL binds to IIa, and partially to Ia, Ib, IIb, and IIIb^38^. McGregor et al. identified human platelet glycoproteins with a high affinity for glycan residues detected by LCA, Con A, and WGA, and no labeling was observed when RCA was applied. LCA revealed 16 bands showing the most heavily labeled gpg, gpIIIa, gpIa, gpIIb, and gpIb. CON A and WGA identified 10 bands with the highest affinity for gpIIIa, gpIV/gpIIIb, gpIIb for CON A, and gpIb for WGA^36^ (Suppl. Table S3).

Abgrall et al. performed a double staining with the gpIIa/gpIIIa complex and lectins (LCA, UEA, LEA, WGA, DBA, SBA, and ECA) and observed a dual positive staining using lectin as a first step and the antibody as a second step^33^. They pointed out that lectins are huge molecules that could interfere with antibody binding and hypothesized that the carbohydrates recognized by these seven lectins are probably not constitutive of the glycoprotein IIb/IIIa complex in addition to the previous characterization by McGregor et al.^36,39^. Tsunehisa et al. also reported that LCA binds preferentially to gpIIb, which interacts with collagen, but also labeled gpIII and IV. Additionally, LCA and PSA inhibited collagen-induced platelet aggregation^40^. Naim et al. observed that PNA, SBA, and RCA induced platelet agglutination; however, when platelets were challenged with vWF and exposed to PNA, agglutination was inhibited^41^. Nurden and Caen^42^, Jenkins et al.^43^, Cooper et al.^44^ previously described gpI as a platelet receptor for vWF, which was also reinforced by Naim et al.. Kawakami and Hirano employed seven lectins (Con A, RCA, WGA, ONA, SBA, DBA, and UEA-1) to describe glycan distribution in human platelet membranes and observed differential affinities to the plasma membrane and open canalicular system (OCS), with Con A, PNA, RCA, and WGA presenting the highest affinity to the plasma membrane, the two former ones being the most intense in the OCS^45^.

Double staining was performed using one of these three lectins (PSA, LCA, and WGA) and vWF, and we could not observe vWF labeling when the antibody was applied together with the lectins as the first or second step in the immunofluorescence, in contrast to a positive vWF expression when analyzed separately. This result not only reinforces the presence of GpIb when megakaryocytes are labeled with LCA and WGA but also demonstrates the presence of its ligand, vWF. Our results should be better explored in the light of Sanjuan-Pla et al. who observed vWF expression shared by a subset of HSC with specific functional properties^46^. vWF was first described as being synthesized by endothelial cells and stored in Weibel-Palade bodies^47,48^. Sporn et al. detected vWF expression in human megakaryocytes in the peripheral blood of a patient with chronic myelogenous leukemia^49^, although a first description was made from guinea pig cells^50^. In our study, we observed some huge megakaryocytes positive for vWF and showing more fragmentation into the nucleus and smaller megakaryocyte negatives; this pattern was also observed when WGA was applied to the fetal liver.

The lectins RCA, WGA and Con A showed specific labeling between E14.5 and E16.5, which may be related to the expression of glycoproteins peculiar to this differentiation stage of megakaryocytes, more frequent at those gestational ages. However, RCA has been shown to recognize galactose residues on the surface of hematopoietic stem cells^51^ and in human adult megakaryocytes^35^. ECL has a pattern among other lectins that recognize galactose and N-acetylglucosamine, as these lectins recognizes Galb4GlcNAc.

Previous studies employing jacalin have shown that lectin is capable of inducing differentiation in the leukemic cell line K562, especially in monocytes^52^. In our study, we identified megakaryocytes from E11.5 − E18.5, liver structures such as capsules and vessels, and other hematopoietic cells, probably from the myeloid lineage when this lectin was applied, and we could not find other previous reports in the literature showing the labeling of this cell by this lectin.

PHA-E and PHA-L showed differences in the intensity of megakaryocyte labeling, as they recognized different oligosaccharides, with a less intense pattern when PHA-L was employed. Another difference concerning the capsule was observed, which was not evident when PHA-L was used, whereas PHA-E recognized some sugar residues in this structure. Darbès et al. confirmed the labeling of PHA-L on neutrophils, megakaryocytes, and granulocytes^34^, whereas PHA-E labeled hematopoietic stem cells were previously reported^51^.

We also observed a rapid increase in the number of megakaryocytic cells between E11.5 and E12.5, which matches the increase in hematopoietic activity reported by Fanni et al. and Ayres-Silva et al. between stages 1 and 2 of hepatic hematopoiesis, with a peak at gestational age E14.5, declining soon after until E17.5, with subtle increases just before birth^10,11^.

It was also possible to note that there was a predominant expression of residues of α-mannose, α-glucose, and GalB3GalNAc in megakaryocytes throughout fetal development, which is of paramount importance for further studies of morphological and lectin-based carbohydrate characterization of the liver environment using immunofluorescence and/or flow cytometry techniques to better understand which sugars mediates hematopoietic cell homing during fetal development. Our results need to be deepened with functional *in vivo* or *in vitro* studies like blocking hematopoietic homing cells. Glycan and lectin arrays will also help to a broad range profile of each hematopoietic and endothelial cell, especially when integrated with morphological information. Knowledge about the similarities and differences in surface sugar residues expressed in hematopoietic and endothelial cells among species remain to be further explored to understand differences in the molecular basis of biological phenomena when using animal models.

Our results showed more integrative data regarding the spatial localization and differentiation stage of megakaryocytes when different lectins were applied. We also reinforce that a more comprehensive study should be carried out using lectins and other traditional markers concomitantly to a better understanding of how megakaryocyte differentiation occurs and their niche interactions in the fetal liver, considering that our results demonstrated glycan diversity in this cell when 21 different lectins were applied. The cellular quantification performed by lectins PSA and LCA demonstrated a more homogenous pattern compared to other lectins applied, since they labeled those cells at all analyzed gestational ages.

The main challenge in lectin-based carbohydrate studies is to specifically know the sugars residues recognized by each lectin. For example, when comparing different lectin datasheets from distinct brands we could find divergent/complementary information about sugar affinity. Sometimes, in the same brand, this information is continuously improving, and more specialized tools for characterization and purification are still under implementation in this field. However, the use of lectins to show megakaryocytes during differentiation is particulary promising as they work very well even when using FFPE samples.

We choose a broad range of lectins for this study to get a comprehensive identification scenario, so more than one lectin recognizing the same sugar residue strengthens the reliability of the study. In fact, it was surprising to observe different intensities of sugar residues being expressed when lectins that were supposed to bind to the same sugar residues showed distinct results. This might be due to the continuous update of lectin sugar residue recognition and different affinities that each lectin have to every residue it bounds.

## Conclusions

It can be concluded that residues of α-mannose, α-glucose, galactose, N-acetylglucosamine, and oligosaccharides (Gal4GlcNAc2Man6 (GlcNAc4) (GlcNAc4Man3) Man4 and Gal4GlcNAc6(GlcNAc2Man3) Man3) are present in megakaryocytes, other hematopoietic cells probably from the myeloid lineage, as well as liver structures such as capsules and vessels, revealed a rich and diverse glycoprotein environment. Mannose and glucose have a wide recognition, with intense and broad expression through the capsule, vessels, and hematopoietic cells, such as megakaryocytes and granulocytes. N-acetylglucosamine has a more specific pattern, and N-acetylgalactosamine is not expressed in fetal hepatic structures or hematopoietic cells.

The findings of the present study suggest that knowledge about the distribution of sugar residues in the fetal liver is of paramount importance for the area since this information is scarce. Most previous results are related to adult human megakaryocytes, platelets and *in vitro* studies. The discovery of specific residues such as galactose and GalB3GalNAc (RCA and Jacalin), and oligosaccharides such as Gal4GlcNAc2Man6 (GlcNAc4) (GlcNAc4Man3) Man4 (PHA-E) in combination with CON A (mannose and glucose) and WGA (N-acetylglucosamine), could be used to constitute a characterization based on the glycoprotein profile of the hematopoietic cell lines, especially megakaryocytes, which should be used to distinguish cells at diverse differentiation processes, such as activate vs. not activated, immature vs. mature, normal vs. neoplastic cells, being a cost-effective alternative. A comprehensive analysis of megakaryocytes in different species is recommended, since the literature review performed in our work showed marked distinctions.

## Materials and methods

### Animals

Swiss Webster mice were placed at night in a micro-ventilated cage to mate at a rate of 1 male to 2 females. The next morning, the female vaginal plug was checked to confirm mating, and mice were then transferred to separate boxes containing wood shavings with food and treated water *ad libitum*. The day of vaginal plug confirmation was determined as gestational age E0.5. At a predetermined gestational age, the animals were anesthetized with a mixture of ketamine and xylazine until death. Fetuses were fixed and processed for paraffin embedding.

### Histological processing

At least one pregnant female with their fetuses (at least 12 pups) at the gestational age of interest (between E11.5 and E18.5) was selected, and their fetuses and fetal livers were collected, fixed in a 3.6% buffered formalin fixative solution modified by Carson^53^ processed into paraffin using a Shandon Citadel 2000 tissue processor (Thermo, USA), and paraffin-embedded (FFPE). Each paraffin block kept a pool of 5-6 fetal livers from the same gestational age from different fetus, or a half-sagital fetus which were cut serially into 5 µm, dewaxed with three successive baths of xylol, hydrated with decreasing ethanol concentrations (3 times with ethanol 100%, once with 95% and 70%), following three washes with distilled water. Serially slides from each block containing 5–6 fetal livers were then subjected to immunofluorescence from all lectins tested, or to hematoxylin-eosin (HE), Sirius Red or Masson’s Trichome stainings. The slides were analyzed by brightfield microscopy using a Axioscope microscope (Zeiss, Germany) equipped with a Axiocam HRc camera (Zeiss, Germany).

### Immunofluorescence

Hydrated slides were immersed in citrate buffer (pH 6.0), for heat-induced Epitope Retrieval (HIER) in a Pascal pressure chamber (Dako, USA) for 3 min at 125 °C. Subsequently, the sections were cooled to room temperature and incubated overnight at 4 °C with Fluorescein-5-isothiocyanate (FITC)-labeled lectins: Con A, LCA, PSA, PNA, APL, ECL, RCA-120, GSL I, PHA-L, PHA-E, Jacalin, sWGA, WGA, DSL, STL, GSL II, LEL, SBA, DBA, VVA, and UEA-1 (Suppl. Table S1). On the following day, the sections were washed three times with phosphate-buffered saline (PBS) and incubated with 1:15000 DAPI (4,6-diamidino-2-phenylindole) for 5 min and washed twice with PBS and mounted with Prolong Gold Antifade (Invitrogen). Other slides were incubated with primary von Willebrand factor (vWF) antibody (1:50) (Cell Marque—250A_RRID-AB_1158309) overnight at 4 °C, and washed three times with phosphate-buffered saline (PBS) on the next day, marked with anti-rabbit AlexaFluor488 secondary antibody (1:750) (A11008_RRID-AB_143165) for 1h at 37 °C, washed three times with PBS, and incubated with DAPI. Negative controls were made using the suppression of primary antibody, when slides were overnight incubated with PBS. In the next day, negative controls were washed three times with PBS and followed the same procedure as tested slides. At least two slides from the same gestational age were analyzed and images are representative from all material investigated. All slides were examined under a confocal laser microscope (LSM 710-ZEISS) equipped with Zen software for analysis and digital image processing.

### Quantification of megakaryocytes

Megakaryocytes were counted using a 40x magnification objective lens in a fluorescence microscope with LED illumination (Colibri, Zeiss). A virtual line was drawn to select the number of fields counted. For gestational ages E11.5 and E12.5, where fetal livers were too small, all fields were considered. For the remaining gestational ages, only odd fields in the virtual line were calculated, and the total number of megakaryocytes in all fields was normalized by the total area counted (each area: 360 × 270 um = 97,200 um^2^). Statistical analyses were performed using the GraphPad program (GraphPad Prism version 5.00 for Windows, San Diego California USA, www.graphpad.com, SCR_002798) using Kruskal-Wallis test with Dunn’s Multiple Comparison pos-test, where a P value <0.05 was considered statistically significant.

### Ethics statement

All procedures involving the use of animals have been approved by Ethics Committee on Animal Experimentation at the Oswaldo Cruz Foundation (Fiocruz) under licence number L029/2015. All methods were performed in accordance with the relevant guidelines and regulations and all the authors complied with the ARRIVE guidelines.

## Supplementary Information

Supplementary Information.

## Data Availability

All materials described in the manuscript, including all relevant raw data, will be freely available to any researcher wishing to use them for non-commercial purposes, without breaching participant confidentiality, when required to the corresponding author.
