# Supplementary material for: Lectin-based carbohydrate profile of megakaryocytes in murine fetal liver during development
Source: Sci Rep. 2023 Apr 25;13:6729. doi: 10.1038/s41598-023-32863-3 (PMC10130079; doi:10.1038/s41598-023-32863-3)
Supplement: Supplementary file 1 — Supplementary Information. [file 41598_2023_32863_MOESM1_ESM.docx]

**Supplementary Information:**

**Lectin-based carbohydrate profile of megakaryocytes in murine fetal liver during development**

Barbara Cristina Marcollino Bomfim^1^, Jessyca Azevedo Silva^1^, Giulia Caminha^1^, João Paulo Rodrigues dos Santos^1^, Marcelo Pelajo-Machado^1,2^, Jackline de Paula Ayres-Silva^1*^

1. Laboratory of Pathology, Oswaldo Cruz Institute - Oswaldo Cruz Foundation (Fiocruz), Rio de Janeiro, Brazil;
2. National Institute of Science and Technology on Neuroimmunomodulation (INCT-NIM), Oswaldo Cruz Institute, Oswaldo Cruz Foundation (Fiocruz), Rio de Janeiro, Brazil.

**Suppl. Table S1 - Lectins used to reveal fetal liver cells and structures.**

| Lectins | Utilized dilutions | Sugar residues | Vector Kits | RRID |
| --- | --- | --- | --- | --- |
| *Concanavalin A* (ConA) | 1:1000 | α-mannose/α-glucose | Kit I FLK - 2100 | AB_2336458 |
| *Lens culinaris* agglutinin (LCA) | 1:1000 | α-mannose/α-glucose | Kit II FLK – 3100 | AB_2336459 |
| *Pisum sativum* agglutinin (PSA) | 1:1000 | α-mannose/α-glucose/ | Kit II FLK – 3100 | AB_2336459 |
| *Arachis hypogaea Agglutinin* (PNA) | 1:400 | Galb3GalNAc | Kit I FLK - 2100 | AB_2336458 |
| *Abrus precatorius* lectin (APL) | 1:50 | galactose | Sigma-Aldrich  L9883 | -------- |
| *Artocarpus integrifolia* (Jackfruit) lectin - Jacalin | 1:500 | GalB3GalNAc | Kit III FLK - 4100 | AB_2336460 |
| *Erythrina cristagalli lectin* (ECL) | 1:200 | Galb4GlcNAc | Kit III FLK - 4100 | AB_2336460 |
| *Ricinus communis* I  Agglutinin (RCA-120) | 1:500 | galactose | Kit I FLK -  2100 | AB_2336458 |
| *Griffonia simplicifolia lectin I* (GSL I) | 1:400 | aGal, aGalNAc | Kit II FLK - 3100 | AB_2336459 |
| *Phaseolus vulgaris -*  Eritroagglutinin (PHA-E) | 1:500 | oligosaccharide ^1^ | Kit II FLK - 3100 | AB_2336459 |
| *Phaseolus vulgaris -*  Leucoagglutinin (PHA-L) | 1:500 | oligosaccharide ^2^ | Kit II FLK - 3100 | AB_2336459 |
| *Datura stramonium* lectin (DSL) | 1:400 | (GlcNAc)2-4 | Kit III FLK - 4100 | AB_2336460 |
| *Triticum vulgaris* (Wheat germ) agglutinin (WGA) | 1:1000 | GlcNAc  (N-acetylglucosamine) | Kit I FLK - 2100 | AB_2336458 |
| Succinylated *Triticum vulgaris* (Wheat germ) agglutinin (sWGA) | 1:500 | GlcNAc  (N-acetylglucosamine) | Kit II FLK - 3100 | AB_2336459 |
| *Solanum tuberosum* lectin (STL) | 1:200 | (GlcNAc)2-4 | Kit III FLK - 4100 | AB_2336460 |
| *Griffonia simplicifolia* lectin II (GSL II) | 1:400 | a or bGlcNAc | Kit III FLK - 4100 | AB_2336460 |
| *Lycopersicon esculentum* lectin (LEL) | 1:200 | (GlcNAc)2-4 | Kit III FLK - 4100 | AB_2336460 |
| *Glycine max* Soybean agglutinin (SBA) | 1:50/1:200 | a>bGalNAc | Kit I FLK – 2100/ Sigma-Aldrich  L1020 | AB_2336458 |
| *Dolichos biflorus* agglutinin (DBA) | 1:200 | aGalNAc | Kit I FLK - 2100 | AB_2336458 |
| *Vicia villosa* agglutinin (VVA) | 1:50 | GalNAc | Kit III FLK - 4100 | AB_2336460 |
| *Ulex europaeus* Agglutinin (UEA I) | 1:50 | aFuc | Kit I FLK - 2100 | AB_2336458 |

1. Legend: Scientific names of the plants from which lectins were extracted, dilutions used, research resource identification portal number (RRID) and sugar residues:1- Galβ4GlcNAcβ2Manα6 (GlcNAcβ4) (GlcNAcβ4Manα3) Manβ4; 2 – Galβ4GlcNAcβ6(GlcNAcβ2Manα3)Manα3; Gal – galactose; NAc- N – acetyl; Glc- glucosamina.

**Suppl. Table S2 – Literature review of platelets and megakaryocytes binding to lectins.**

| Year of publication |  | 1974(35) | 1977(38) | 1979(39) | 1985(22) | 1985 | 1991(33) | 1991 | 1993(34) | 1993 | 1993 | 1993 |
| --- | --- | --- | --- | --- | --- | --- | --- | --- | --- | --- | --- | --- |
| Specie |  | H | H | H | GP | GP | H | H | C | C | D | D |
| Platelets |  | X |  | X | X |  | X |  | X |  | X |  |
| Megakaryocyte |  |  | X |  |  | X |  | X |  | X |  | X |
| Sugar specificity | lectin |  |  |  |  |  |  |  |  |  |  |  |
| Galactose, Complex structures | PHA-L | NT | NT | NT | NT | NT | NT | NT | + | NEG | + | + |
| Galactose, N-Acetylgalactosamine, Lactose | ECL | NT | NT | NT | NT | NT | + | +/- | NT | NT | NT | NT |
| Galactose/ N-Acetylgalactosamine | RCA | + | NT | NEG | NT | NT | NT | NT | NEG | NEG | NEG | NEG |
| Galactose/ N-Acetylgalactosamine | SBA | NEG | NT | NT | NT | NT | NEG | + | NEG | NEG | NEG | NEG |
| N-Acetylgalactosamine | DBA | NT | NT | NT | NT | NT | NEG | +/- | NT | NT | NT | NT |
| Galactose | ABA | + | NT | NT | NT | NT | NT | NT | NT | NT | NT | NT |
| Galactose | APL | NT | + | NT | NT | NT | NT | NT | NT | NT | NT | NT |
| Galactose | PNA | NT | NT | NT | NT | NT | NT | NT | NEG | + | NEG | + |
| Mannose e Glucose | CON A | + | NT | + | NT | NT | NT | NT | NEG | NEG | NEG | NEG |
| Mannose e Glucose | LCA | NEG | + | + | NT | NT | NT | + | + | NEG | + | + |
| Mannose e Glucose | LSA | NEG | NT | NT | NT | NT | NT | NT | NT | NT | NT | NT |
| Mannose e Glucose | PSA | NEG | NT | NT | NT | NT | NT | NT | + | NEG | + | + |
| N-Acetylglucosamine | LEL | NT | NT | NT | NT | NT | + | + | NT | NT | NT | NT |
| N-Acetylglucosamine | WGA | + | + | + | + | + | + | + | NT | NEG | + | + |
| N-Acetylgalactosamine(54)/ glucosamine(55) | PCA | + | NT | NT | NT | NT | NT | NT | NT | NT | NT | NT |
| Fucose, arabinose | UEA | NT | NT | NT | NT | NT | + | + | NEG | NEG | NEG | NEG |

Legend: H- Human; GP- guinea pig; C- cat; D- dog; + positive; Neg- negative; +/- some were positive and some negative; NT, not tested. (54) - Feria et al., 1996; (55) - Pan & NG, 2015; (35) - Greenberg & Jamieson; (38) - Clemetson et al. ; (39) - McGregor et al; (22) - Schick and Filmyer; (33) - Abgrall et al; (34) - Darbès et al.

**Suppl. Table S3 – Platelets glycoproteins and their lectin binding** (data obtained from (33) Abgrall et al., 1991; (38) - Clemetson et al., 1977; (35) - Greenberg and Jamieson, 1974; (39) – McGregor et al., 1979; (37) - Nachman et al., 1973).

| Glycoproteins (GP) | I |  | IIa | IIa+IIb | III |  |  |
| --- | --- | --- | --- | --- | --- | --- | --- |
|  | Ia | Ib | IIa | IIb | IIIa | IIIb | IV |
| APL (38) | + | + | + | + |  | + |  |
| CON A (39, 37) | + |  |  | + | + | + | + |
| LCA(38) | + | + |  | + | + |  |  |
| WGA (33,38, 35) | + | + |  | + | + |  |  |
| PCA (35) |  |  | + |  |  |  |  |
| LEA (33) |  |  |  | + | + |  |  |
| UEA-1 (33) |  |  |  | + | + |  |  |
| DBA (33) |  |  |  | + | + |  |  |
| SBA (33) |  |  |  | + | + |  |  |
| ECA (33) |  |  |  | + | + |  |  |

Supplementary figure S4


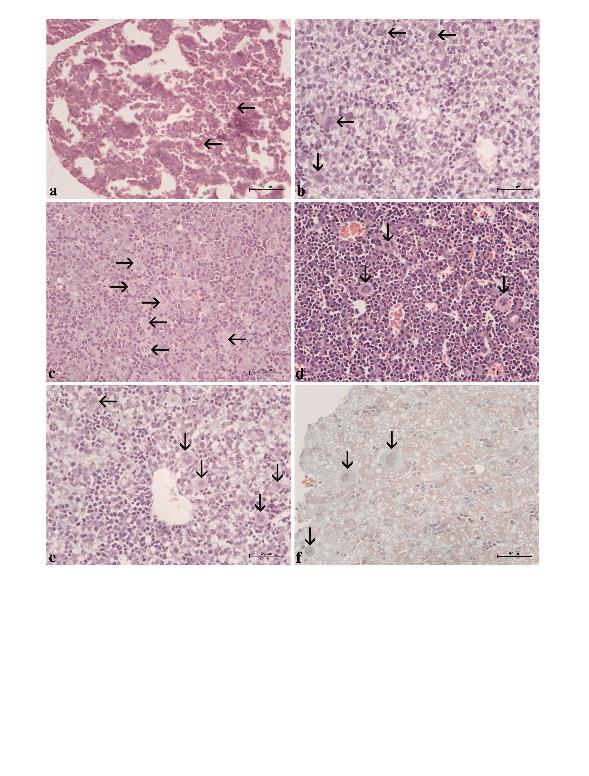


Figure S4: Morphological complexion of megakaryocytes during fetal liver development. At 11.5 (a) the fetal liver is small, and filled with hematopoietic cells, mainly of erythrocytic lineage, with some megakaryocytes (→). At 12.5 (b), the fetal liver is slightly bigger, showing clusters of erythroid differentiation, presenting megakaryocytes at distinct differentiation stages. Hepatocytes are metabolically active by the presence of products in their cytoplasm, represented by white spaces. At 13.5 (c), fetal livers are filled with hematopoietic cells, without visible spaces between hepatocytes and hematopoietic niches. Many stages of megakaryocytes are proliferating. At 14.5 (d), the fetal liver is two to three times bigger than in the previous day, with a similar hematopoietic differentiation aspect Some sinusoidal spaces are visible. At 16.5 (e) less hematopoietic cells are observed in the fetal liver, as they are homing to the bone marrow, however, megakaryocytes are still seen. At the day of birth (0 day pos partum), hepatocytes are metabolically active, with many vacuolization, along with some clusters of erythroid cells. Many stages of megakaryocytes persist differentiating in the liver. A, C, D – HE; B, E - Sirius Red; F – Masson’s Trichome.
